# Trends in COVID-19 Vaccination Intent, Determinants and Reasons for Vaccine Hesitancy: Results from Repeated Cross-Sectional Surveys in the Adult General Population of Greece during November 2020–June 2021

**DOI:** 10.3390/vaccines10030470

**Published:** 2022-03-18

**Authors:** Vana Sypsa, Sotirios Roussos, Vasiliki Engeli, Dimitrios Paraskevis, Sotirios Tsiodras, Angelos Hatzakis

**Affiliations:** 1 Department of Hygiene, Epidemiology and Medical Statistics, Medical School, National and Kapodistrian University of Athens, 11527 Athens, Greece; sotirisroussos@yahoo.co.uk (S.R.); vasia9596@gmail.com (V.E.); dparask@med.uoa.gr (D.P.); ahatzak@med.uoa.gr (A.H.); 2 4th Department of Internal Medicine, Medical School, National and Kapodistrian University of Athens, 12462 Athens, Greece; sotirios.tsiodras@gmail.com

**Keywords:** COVID-19, vaccination intention, vaccination hesitancy, repeated cross-sectional surveys

## Abstract

Vaccine hesitancy is a major barrier to achieving large-scale COVID-19 vaccination. We report trends in vaccination intention and associated determinants from surveys in the adult general population in Greece. Four cross-sectional phone surveys were conducted in November 2020 and February, April and May 2021 on nationally representative samples of adults in Greece. Multinomial logistic regression was used on the combined data of the surveys to evaluate independent predictors of vaccination unwillingness/uncertainty. Vaccination intention increased from 67.6% in November 2020 to 84.8% in May 2021. Individuals aged 65 years or older were more willing to be vaccinated (May 2021: 92.9% vs. 79.5% in 18–39 years, *p* < 0.001) but between age-groups differences decreased over time. Vaccination intention increased substantially in both men and women, though earlier among men, and was higher in individuals with prograduate education (May 2021: 91.3% vs. 84.0% up to junior high). From multivariable analysis, unwillingness and/or uncertainty to be vaccinated was associated with younger age, female gender (in particular in the April 2021 survey), lower educational level and living with a child ≤12 years old. Among those with vaccine hesitancy, concerns about vaccine effectiveness declined over time (21.6% in November 2020 vs. 9.6% in May 2021, *p* = 0.014) and were reported more often by men; safety concerns remained stable over time (66.3% in November 2020 vs. 62.1% in May 2021, *p* = 0.658) and were reported more often by women. In conclusion, vaccination intention increased substantially over time. Tailored communication is needed to address vaccine hesitancy and concerns regarding vaccine safety.

## 1. Introduction

Severe acute respiratory syndrome coronavirus 2 (SARS-CoV-2) has spread worldwide, and had caused more than 172 million cases and 3.7 million deaths by early June 2021 [1]. Non-pharmaceutical interventions have been employed worldwide to reduce COVID-19 healthcare demand and mortality. These interventions constitute a short-term solution as their implementation over long periods has a major economic and social impact; in addition, the hardship associated with these measures has resulted in “pandemic fatigue”, i.e., in the gradual demotivation of people to follow recommended protective behaviours [2]. The continuing transmission of SARS-CoV-2 gives rise to new variants that may be more transmissible or more resistant to existing immunity and/or more pathogenic. As a result, developing safe and effective vaccines and achieving large-scale immunisation is critical. Several vaccines have been approved by national regulatory authorities and more are under review [3]. High vaccination rates will be necessary to reduce transmission and the healthcare burden, in particular with the emergence of more transmissible variants [4]. Apart from supply, administration and demand constraints that stand in the way of achieving high vaccination coverage rapidly [5], vaccine hesitancy is an important additional problem to be addressed.

The SAGE Working Group on Vaccine Hesitancy defined vaccine hesitancy as “the delay in acceptance or refusal of vaccination despite availability of vaccination services” [6]. The “3 Cs” model highlights concepts influencing the decision to be vaccinated such as Complacency, Convenience/Constraints and Confidence [6]. Complacency exists when the perceived risk from the diseases is low and, thus, vaccination is not deemed a necessary action. Convenience relates to practical barriers, such as geographical accessibility and physical availability. Confidence relates to the trust in the effectiveness and safety of vaccines, the system that delivers them and the motivations of policy-makers who decide on the vaccines needed [6,7]. Additional indicators have been proposed, such as calculation (engagement in extensive information searching) and collective responsibility (willingness to protect others) (5 Cs model) [8]. Reluctance to accept immunisation is not a new problem but during the COVID-19 pandemic it is more urgent than ever. The fact that COVID-19 vaccines were developed rapidly and that some of them were based on a highly innovative approach may have worsened vaccine hesitancy [9]. Given the high levels of vaccination that are needed, vaccine hesitancy poses a significant risk to minimising the effects of the COVID-19 pandemic. Assessing the trends of vaccination intention over time and exploring its determinants could be of help to optimise communication and increase willingness to be vaccinated.

Our aim was to examine the trends in vaccination intention over time, and analyse determinants associated with vaccination hesitancy based on data from four cross-sectional surveys on nationally representative samples of adults in Greece. The surveys were implemented during the pandemic, from a period where vaccines were not yet available (November 2020) until approximately 5.5 million doses were distributed in the country’s population (May 2021).

## 2. Materials and Methods

### 2.1. Phone Surveys and Questionnaire

We conducted four cross-sectional phone surveys with independent samples in the following periods: 17 November–3 December 2020, 1 February–18 February 2021, 1 April–12 April 2021 and 17 May–5 June 2021. In each survey, we recruited approximately 1200 persons of all ages using proportional quota sampling based on age and sex from urban, semi-urban and rural areas in Greece. Questions concerning vaccination intention were asked only to adult respondents. Trained staff administered the questionnaires by phone.

The questionnaire included sections on sociodemographic characteristics (age, gender, education, occupation, nationality, area, household size, age of household members), social contacts, previous SARS-CoV-2 testing, vaccination intention and reasons for not intending to be vaccinated. Respondents were asked whether they would consider being vaccinated—when it was their turn according to the national vaccination plan—with the following response categories: “Definitely yes”, “Probably yes”, “Probably not”, “Definitely not”, “Don’t know”, “Already vaccinated”. Those who responded “Probably not”/“Definitely not” were asked to cite one or more reasons for not being willing to be vaccinated.

Vaccination roll-out in Greece is organised according to age. Other groups, such as healthcare workers and persons with underlying medical conditions, are also prioritised. In the periods when the surveys took place, vaccine availability was as follows: November 2020—no vaccine; February 2021—health care workers and persons aged 80 years or older; first half of April—individuals aged 60 years or older; second half of May—persons aged 30 years or older as well as 16–29 years old with underlying medical conditions (on 29th May, vaccination was available to all adults).

### 2.2. Statistical Methods

We estimated the proportions in each category of vaccination intention and the corresponding 95% confidence intervals (95% CI) after standardising for the age and sex distribution of the adult population in Greece.

We further assessed vaccination intention (“probably/definitely yes” or “already vaccinated”, “probably/definitely not”, “don’t know”) according to respondents’ characteristics and performed comparisons using the chi-squared test.

A multinomial logistic regression model was used on the combined data of the four surveys to evaluate independent predictors of vaccination unwillingness and uncertainty. The outcome variable in this model was coded such that those likely to vaccinate (“probably/definitely yes” or “already vaccinated”) were compared to those who were (i) unlikely to be vaccinated (“probably/definitely not”) and (ii) undecided about whether to be vaccinated (“Don’t know”). A model containing all variables whose univariate test had a *p*-value less than 0.25 was considered. Then, variables that did not contribute to the model, based on their Wald statistic, were eliminated and the new model was compared to the old through the likelihood ratio statistic. Variables whose exclusion gave a non-significant likelihood ratio statistic were omitted from the model. Regression coefficients were exponentiated and are presented as relative risk ratios (RRRs) with corresponding 95% confidence intervals (95% CIs). We included interaction effects to assess changes in the impact of explanatory variables over time as well as to evaluate whether the impact of the variables was moderated by other characteristics of the respondents. The goodness-of-fit was assessed using a generalised Hosmer–Lemeshow goodness-of-fit test for multinomial logistic regression models [10]. The analysis was performed using the STATA statistical software program (STATA version 14.0; StataCorp, College Station, TX, USA).

### 2.3. Ethical Issues

The protocol of the surveys was approved by the Institutional Review Board of the Hellenic Scientific Committee for the Study of AIDS and STDs (16 November 2020). Participants provided oral informed consent.

## 3. Results

### 3.1. Characteristics of the Participants

In the four surveys, individuals 18–39, 40–64 and 65+ years old constituted 25.5–30.0%, 38.1–45.9% and 28.2–31.9% of the sample, respectively (Table 1).

Approximately 53–59% were women and 46–53% had completed university or postgraduate studies. The median household size in the surveys was 2–3 persons; approximately one third and one fifth of the respondents lived in the same household with a person ≥65 years and a child ≤12 years old, respectively. There were statistically significant differences in these characteristics between the four surveys.

### 3.2. Trends in Vaccination Intention

**Figure 1:**
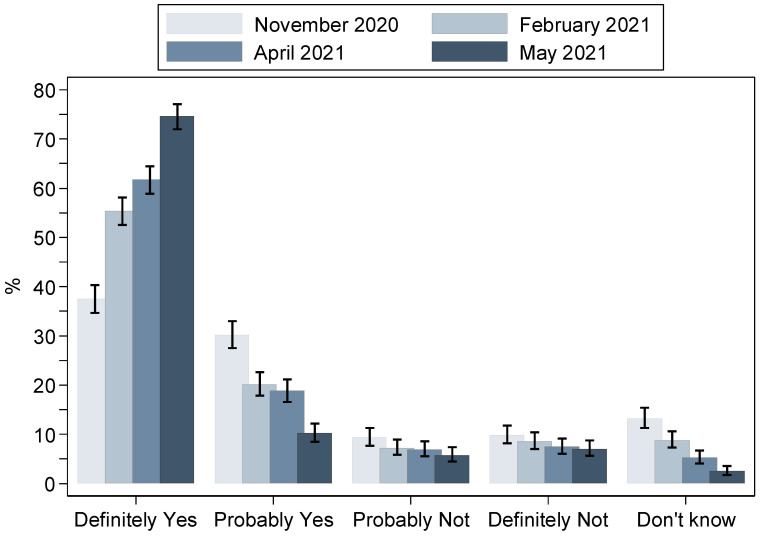
Responses concerning COVID-19 vaccination intention in four cross-sectional surveys implemented in the adult population of Greece during the pandemic (proportions and 95% confidence intervals). The category “Definitely yes” includes also those already vaccinated. The proportions are standardised to the age and sex distribution of the adult population in Greece.

The trends in vaccination intention are depicted in Figure 1. There was a statistically significant increase over time in the proportion of those answering “Definitely yes” (including those already vaccinated) from 37.4% in November 2020 to 74.6% in May 2021.

Across surveys, a decrease was observed in the proportion reporting “Probably yes” (from 30.2% to 10.2%) or “Don’t know” (from 13.2% to 2.5%) and a moderate reduction in those reporting “Probably not” (from 9.3% to 5.7%). Conversely, the proportion of participants answering “Definitely not” remained relatively stable over time (9.9% in November 2020 and 7.0% in May 2021).

### 3.3. Trends in Vaccination Intention According to Respondents’ Characteristics

In November 2020, intent to receive the COVID-19 vaccine (“definitely/probably yes”) was lower in younger individuals (58.7%, 68.9% and 79.1% in 18–39, 40–64 and 65+ years old, respectively, *p* < 0.001) (Table 2). Over time, there was an increase in willingness to be vaccinated in all age groups and, although these differences were narrowed, they were still statistically significant in May 2021 (79.5%, 85.4% and 92.9% in 18–39, 40–64 and 65+ years old, respectively, *p* < 0.001).

In November 2020, similar percentages in vaccination intent were identified between men and women (69.8% and 68.5%, respectively, *p* = 0.821) and across educational levels (up to junior high: 66.5%, high school: 70.8%, university: 68.5%, postgraduate education: 72.1%, *p* = 0.670). Over time, willingness to be vaccinated increased substantially in men and women, though earlier among men, and vaccination intention was similar in the most recent survey of May 2021 (85.7% of men and 86.1% of women) (Table 2). Vaccination intention increased substantially in all educational levels but this increase was more pronounced in university graduates or those with postgraduate qualifications (May 2021: 84.0%, 83.5%, 87.3% and 91.3% for up to junior high, high school, university and postgraduate, respectively, *p* = 0.036). 

In all surveys, respondents who lived in the same household with a person 65 years or older were more likely to report willingness to be vaccinated as compared to those living alone or with younger individuals. This difference was narrowed in the most recent survey but remained statistically significant (May 2021: 88.7% vs. 84.6%, respectively, *p* = 0.053). In the first survey in particular, where individuals 18–39 years old had substantially lower vaccination intention compared to other groups, young individuals living with an older person were more likely to be willing or indecisive as compared to those not living with an older person (83.3% vs. 70.1%, *p* = 0.021).

Living with a child ≤12 years old was associated with higher vaccination refusal in the first surveys (November: 26.6% vs. 16.7%, February: 21.2% vs. 12.7%) but there was no difference identified in the most recent surveys in April and May 2021. A closer look at the data revealed that this finding was evident among female respondents. In November 2020, for example, vaccination refusal for those living with a child vs. those without was 21.3% vs. 17.5% for men (*p* = 0.180) and 31.1 vs. 16.1% (*p* < 0.001) for women.

### 3.4. Predictors of Unwillingness to Be Vaccinated or Uncertainty with Regard to Vaccination

The results from the multinomial logistic regression model for the determinants of unwillingness or uncertainty about being vaccinated are shown in Table 3. 

The period when the survey took place had an effect with decreased vaccine hesitancy over time; compared to May 2021, respondents in November 2020 were more likely to be unwilling or uncertain and respondents in February 2021 were more likely to be uncertain. Groups at increased risk for unwillingness and uncertainty to be vaccinated against COVID-19 were younger individuals (18–39 vs. 65+: unwilling: RRR = 7.1; 95% CI: 4.6 to 11.1, uncertain: RRR = 4.0; 95% CI: 2.4 to 6.6 and 40–64 vs. 65+: unwilling: RRR = 4.3; 95% CI: 2.8 to 6.7, uncertain: RRR = 2.5; 95% CI: 1.5 to 4.1) as well as individuals with lower educational levels (up to junior high vs. postgraduate: unwilling: RRR = 2.7; 95% CI: 1.9 to 3.9, uncertain: RRR = 4.3; 95% CI: 2.5 to 7.4, high school vs. postgraduate: unwilling: RRR = 2.1; 95% CI: 1.5 to 3.0, uncertain: RRR = 2.6; 95% CI: 1.5 to 4.3, university vs. postgraduate: unwilling: RRR = 1.8; 95% CI: 1.3 to 2.5, uncertain: RRR = 1.6; 95% CI: 0.9 to 2.7).

**Figure 2:**
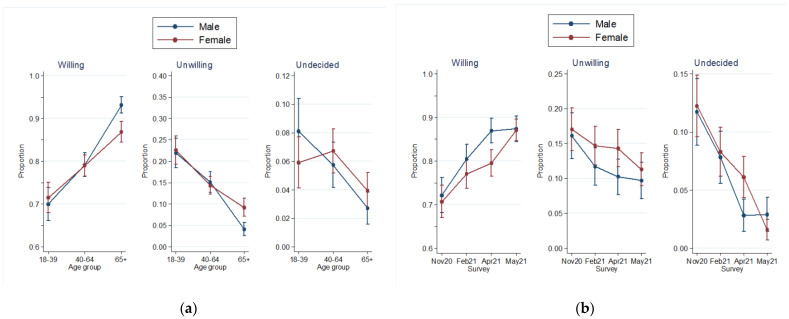
Adjusted predictions of willingness, unwillingness and uncertainty about being vaccinated: (**a**) effect of age according to the gender of respondents; (**b**) effect of survey period according to the gender of respondents.

Women were more likely to be unwilling as compared to men; this finding was more apparent among women aged 65 years or older (Figure 2a). Living with a child ≤12 years old was not found to be related to uncertainty around the COVID-19 vaccine but was associated with unwillingness (RRR vs. no child at home: 1.3; 95% CI: 1.1 to 1.6). There was an interaction between gender and survey period with women experiencing lower willingness and higher uncertainty in April 2021 as compared to men (Figure 2b).

### 3.5. Reasons for Not Intending to Be Vaccinated

The reasons most frequently cited for not intending to be vaccinated were concerns about the safety and the effectiveness of the vaccine (65.5% and 15.7%, respectively). A larger percentage of the February, April and May 2021 survey participants than November 2020 participants reported effectiveness concerns (21.6%, 17.1% and 15.0% vs. 9.6%, respectively, *p* = 0.014). Safety concerns were reported at similar percentages in all four periods (66.3%, 62.6%, 67.7% and 62.1% in November 2020, February, April and May 2021, *p* = 0.658). Some respondents specifically cited allergies as a concern relating to the safety of the vaccine at the beginning of the vaccination process in the country but this concern diminished as vaccination progressed (fear of allergies among unwilling/uncertain: 0%, 7.6%, 2.4% and 0.7% in November 2020, February, April and May 2021, respectively, *p* < 0.001).

Men more often reported doubts in vaccine effectiveness (19.9% vs. 12.4% reported by women, *p* = 0.007) whereas women reported at higher percentages concerns about safety as compared to men (67.8% vs. 60.8%, *p* = 0.062). The latter finding was modified by the age of the respondents; doubts about safety were reported more often by women aged 40–64 years old as compared to men and this finding was independent of the educational level and the presence of a child in the household (Supplementary Figure S1).

As to other reasons for not intending to be vaccinated, 8.6% reported that they considered themselves as not being at risk of getting COVID-19 and 5.0% that COVID-19 is a mild disease.

## 4. Discussion

We have provided estimates on vaccination intention from four surveys conducted in the general adult population in Greece before the initiation of vaccination as well as during vaccination roll-out. A literature review on vaccination intention by October 2020 has found a substantial variation in vaccination intention between countries before vaccination was available [11]. Our estimate of 67.6% in November 2020 suggests that vaccination intention in the general adult population in Greece was high. It compares to a range of estimates between 59–70% in US, Australia, Finland and UK from surveys performed between July–December 2020 [12,13,14,15,16] and lies between the estimates of 71.5% and 54% reported by surveys performed in 19 and 15 countries in June 2020 and January 2021, respectively [17,18]. There is one study assessing vaccination intention in a nationwide sample of the adult population in Greece early in the pandemic (April–May 2020) with similar findings to those reported in our survey of November 2020 (57.7% were willing, 26.0% were unwilling and 16.3% were undecided) [19]. A survey performed in September–October 2020 in the personnel of healthcare facilities in Greece reported lower vaccination intention (51.1%) [20] whereas another conducted in December 2020 among healthcare workers reported a higher vaccination intention (78.5%) [21]. However, comparisons with studies among healthcare workers should also take into account that these samples have a different age and sex distribution than that of the general adult population.

Vaccination intention in the most recent survey conducted in May 2021 increased to 84.8%. By that time, persons aged 30 years or older as well as 16–29 years old with underlying medical conditions had access to vaccination and, at the end of May, vaccination was offered to all adults. On 31st May 2021, approximately 19% of the Greek population (23% of adults) was fully vaccinated. The most striking change from November 2020 to May 2021 was the 2-fold increase in those who were certain to receive vaccination. This increase seems to result from a shift in those who were initially positive towards vaccination (“Probably yes”) or undecided, as these proportions declined substantially over time; interestingly, the proportion of participants answering “Definitely not” remained relatively stable over time and at low numbers. Although willingness to be vaccinated among adults in May 2021 was high, this does not ensure that high vaccination rates can be reached. Vaccination intention is likely to be greater than actual vaccine uptake due to an “intention-behaviour gap” [22]. Despite that, the high vaccination intention observed in our survey among individuals 65 years and older (92.9%) is promising concerning achieving the target of reducing morbidity, mortality and healthcare burden. By 2nd March 2022, 90.7% of those aged 60 years or older in Greece had received at least one vaccine dose [23].

In November 2020, i.e., before the initiation of vaccination, willingness to be vaccinated did not differ according to educational level or gender; it was higher in older individuals and people living with the elderly and lower in people living with a child. Subsequently, an increase in vaccination intention was apparent in all age groups and, in particular, among individuals 18–39 years old; as a result, differences according to age were narrowed over time but were not eliminated as people 65 years or older were still more willing to be vaccinated. Apart from younger age, women were more likely to be unwilling or uncertain about being vaccinated and that was independent of educational level. This finding has been reported in the literature [12,13,15,24,25]. We have identified effect modification of the relationship between age and vaccination intention by gender; women 65 years or older were less likely to accept vaccination compared to males. In addition, women exhibited higher uncertainty during vaccination roll-out (in particular in the survey of April 2021) and that was independent of their educational level and their age. This was most probably caused by information on vaccine-linked blood clots being announced by the European Medicines Agency in early April [26]. The finding on the impact of gender deserves attention as, apart from the fact that women constitute half of the world population and account for a large proportion of healthcare workers [27], they also influence healthcare decisions in their families.

Higher unwillingness and uncertainty were also identified among respondents with lower educational levels. Educational levels, as well as related social characteristics, such as income, have also been found to affect attitudes towards COVID-19 vaccination elsewhere [13,14,24,28,29]. In the US in particular, based on the data from the “Understanding Coronavirus in America” survey, it became evident that educational level played a greater role in people’s willingness to be vaccinated than race and ethnicity [30]. This finding shows that people with lower educational levels constitute a group that cannot be easily convinced by conventional risk communication and additional effort is needed. We have also found that living with a child ≤12 years old was associated with higher probability of vaccine refusal. This has been reported in another study as well [13]. Probably, parents who are not convinced about the safety of the vaccine are more conservative in taking a potential risk.

Based on our findings, there is a core of people who are not convinced about the effectiveness and the safety of the vaccine and the size of this population—in particular those refusing vaccination—did not change substantially during the expansion of vaccination around the world. In our studies, approximately two-thirds of those not intending to be vaccinated reported as main reason concerns about the safety of the vaccine and 16% about its effectiveness. Although doubts about vaccine effectiveness decreased over time, safety concerns remained at similarly high levels. Similar findings were observed among vaccine-hesitant Italian university students during March–May 2021 [31]. It is of note that we identified gender differences with men reporting more often doubts in vaccine effectiveness and women reporting at higher percentages concerns about safety. This explains why, although men and women did not differ in their vaccination intention in November, over time men became more quickly convinced and were more willing to be vaccinated whereas women were more often negative or undecided.

Our study has some limitations. First, selection bias could exist in the phone surveys. Interviewers informed the persons who picked up the phone that they were going to ask questions about COVID-19. It is possible that people with negative attitudes towards the pandemic would be more likely to decline. Second, responses might be affected by social desirability bias; for example, respondents might answer questions about vaccination intention in a manner that would be viewed favourably by the interviewers. Both these biases might result in overestimating vaccination intention. However, the findings concerning trends in vaccination intention over time and associated determinants should not be affected by this limitation. Third, the findings on vaccination intention over time and associated determinants could be influenced by the disease burden in the country, social distancing measures or media coverage during the specific survey period. This is not necessarily a limitation as, for example, it allowed us to assess the impact of news on vaccine-linked blood clots on vaccination intention among women. Finally, there were statistically significant differences in sociodemographic variables of the participants in the four surveys. However, vaccination intention/hesitancy in each period was estimated after standardising to the age and sex distribution of the adult population in Greece. In addition, the model on predictors of unwillingness and uncertainty to vaccinate also adjusts for these variables. 

A strength of this study is that vaccination intention and its determinants have been evaluated through four repeated cross-sectional surveys implemented over a period extending from before vaccination was available until it was offered to all the adults in the country. 

## 5. Conclusions

To our knowledge, this is the first study assessing vaccination intention in a nationwide sample of adults in Greece at multiple time points during the pandemic, before vaccination was available and during vaccination roll-out. Vaccination intention increased from 67.6% in November 2020 to 84.8% in May 2021 and unwillingness and/or uncertainty to be vaccinated was associated with younger age, female gender (in particular in the April 2021 survey), lower educational level and living with a child ≤12 years old. As persons aged 18 years or older constitute approximately 83% of the population in Greece, a vaccination intention of 84% could result in 69% of the total population accepting the vaccine. To ensure that we reach this target, as well as the uptake of booster doses according to recommendations, it is important to address convenience and constraints for those willing to be vaccinated by removing practical barriers to vaccination and improving ease of access in order to close the potential “intention-behaviour gap”. A major concept influencing the decision to accept the COVID-19 vaccine is confidence; people who refuse or are uncertain about being vaccinated usually express concerns about the safety of the vaccine. Tailored communication that takes into account the characteristics of the population with lower vaccine acceptance and providing consistent and accurate information are needed to address vaccine hesitancy and safeguard the public against vaccine misinformation.

## Figures and Tables

**Table 1:** Socio-demographic characteristics of the participants in the 4 phone surveys in Greece.

Participants’ Characteristics	17 November– 3 December 2020 (N = 1097)	1–18 February 2021 (N = 1196)	1–12 April 2021 (N = 1189)	17 May–5 June 2021 (N = 1200)
Age, *n* (%)				
18–39	329 (30.0)	305 (25.5)	305 (25.7)	322 (26.8)
40–64	418 (38.1)	549 (45.9)	545 (45.8)	540 (45.0)
65+	350 (31.9)	342 (28.6)	339 (28.5)	338 (28.2)
Gender, *n* (%)				
Male	504 (45.9)	562 (47.0)	524 (44.1)	495 (41.3)
Female	593 (54.1)	634 (53.0)	665 (55.9)	705 (58.8)
Educational level, *n* (%)				
Up to junior high school	221 (20.2)	239 (20.1)	233 (19.7)	188 (15.8)
Up to general high school	370 (33.7)	339 (28.4)	326 (27.5)	369 (30.9)
University	438 (39.9)	463 (38.8)	498 (42.0)	487 (40.8)
Postgraduate education	68 (6.2)	151 (12.7)	128 (10.8)	149 (12.5)
Household size, median (25th, 75th)	2 (2, 3)	3 (2, 4)	3 (2, 4)	3 (2, 4)
Living with a person ≥65 years old, *n* (%)	395 (36.0)	366 (30.6)	376 (31.6)	390 (32.5)
Living with a child ≤12 years old, *n* (%)	158 (14.4)	227 (19.0)	235 (19.8)	194 (16.2)

**Table 2:** Vaccination intention (% [95% confidence interval]) over time by respondents’ characteristics (Willing: “Probably/Definitely yes” or “Already vaccinated”, Unwilling: “Probably/Definitely not”, Undecided: “Don’t know”).

Participants’ Characteristics	17 November–3 December 2020			1–18 February 2021			1–12 April 2021			17 May–5 June 2021		
	(N = 1097)			(N = 1196)			(N = 1189)			(N = 1200)		
	Willing	Unwilling	Undecided	Willing	Unwilling	Undecided	Willing	Unwilling	Undecided	Willing	Unwilling	Undecided
Age group												
18–39	58.7 [53.2, 63.9]	26.7 [22.2, 31.8]	14.6 [11.2, 18.8]	66.9 [61.4, 72.0]	23.6 [19.2, 28.7]	9.5 [6.7, 13.4]	75.7 [70.6, 80.2]	18.0 [14.1, 22.8]	6.2 [4.0, 9.6]	79.5 [74.7, 83.6]	18.6 [14.7, 23.3]	1.9 [0.8, 4.1]
40–64	68.9 [64.3, 73.2]	17.9 [14.5, 21.9]	13.2 [10.2, 16.8]	76.3 [72.6, 79.7]	14.4 [11.7, 17.6]	9.3 [7.1, 12.0]	79.6 [76.0, 82.8]	15.0 [12.3, 18.3]	5.3 [3.7, 7.6]	85.4 [82.1, 88.1]	11.1 [8.7, 14.1]	3.5 [2.3, 5.5]
65+	79.1 [74.6, 83.1]	10.3 [7.5, 13.9]	10.6 [7.8, 14.3]	87.1 [83.1, 90.3]	5.8 [3.8, 8.9]	7.0 [4.7, 10.3]	88.2 [84.3, 91.2]	8.0 [5.5, 11.4]	3.8 [2.2, 6.5]	92.9 [89.6, 95.2]	5.9 [3.8, 9.0]	1.2 [0.4, 3.1]
Gender												
Male	69.8 [65.7, 73.7]	18.1 [14.9, 21.7]	12.1 [9.5, 15.3]	78.5 [74.9, 81.7]	13.3 [10.8, 16.4]	8.2 [6.2, 10.8]	84.4 [81.0, 87.2]	12.4 [9.8, 15.5]	3.2 [2.0, 5.2]	85.7 [82.3, 88.5]	11.1 [8.6, 14.2]	3.2 [2.0, 5.2]
Female	68.5 [64.6, 72.1]	18.2 [15.3, 21.5]	13.3 [10.8, 16.3]	75.7 [72.2, 78.9]	15.1 [12.6, 18.2]	9.1 [7.1, 11.7]	78.5 [75.2, 81.5]	14.9 [12.4, 17.8]	6.6 [5.0, 8.8]	86.1 [83.3, 88.5]	12.1 [9.8, 14.7]	1.8 [1.1, 3.2]
Educational level												
Up to junior high school	66.5 [60.0, 72.4]	15.4 [11.2, 20.8]	18.1 [13.5, 23.8]	71.1 [65.0, 76.5]	15.5 [11.4, 20.7]	13.4 [9.6, 18.3]	78.1 [72.3, 83.0]	13.7 [9.9, 18.8]	8.2 [5.3, 12.4]	84.0 [78.1, 88.6]	12.8 [8.7, 18.4]	3.2 [1.4, 6.9]
Up to high school	70.8 [66.0, 75.2]	15.9 [12.6, 20.0]	13.2 [10.1, 17.1]	72.6 [67.6, 77.1]	17.1 [13.5, 21.5]	10.3 [7.5, 14.1]	77.9 [73.1, 82.1]	16.3 [12.6, 20.7]	5.8 [3.7, 9.0]	83.5 [79.3, 86.9]	13.8 [10.7, 17.7]	2.7 [1.5, 5.0]
University	68.5 [64.0, 72.7]	20.8 [17.2, 24.8]	10.7 [8.2, 14.0]	81.0 [77.2, 84.3]	13.4 [10.6, 16.8]	5.6 [3.8, 8.1]	81.9 [78.3, 85.1]	13.9 [11.1, 17.2]	4.2 [2.8, 6.4]	87.3 [84.0, 90.0]	11.1 [8.6, 14.2]	1.6 [0.8, 3.3]
Postgraduate education	72.1 [60.2, 81.5]	22.1 [13.7, 33.6]	5.9 [2.2, 14.8]	84.1 [77.3, 89.1]	9.3 [5.6, 15.1]	6.6 [3.6, 11.9]	91.4 [85.1, 95.2]	7.8 [4.2, 14.0]	0.8 [0.1, 5.4]	91.3 [85.5, 94.9]	6.7 [3.6, 12.1]	2.0 [0.6, 6.1]

**Table 3:** Predictors of unwillingness and uncertainty to be vaccinated against COVID-19 based on the data collected from the four cross-sectional surveys in Greece (results from multivariable analysis, N = 4682 participants).

Variables	Unwilling		Undecided	
	RRR ^†^ [95% CI]	*p*	RRR ^†^ [95% CI]	*p*
Gender Female/Male	2.2 [1.3, 3.9]	0.006	0.8 [0.3, 1.9]	0.571
Period				
April 2021/May 2021	1.1 [0.7, 1.6]	0.778	1.0 [0.5, 2.0]	0.947
February 2021/May 2021	1.3 [0.9, 1.9]	0.170	2.9 [1.6, 5.4]	<0.001
November 2020/May 2021	2.0 [1.4, 2.9]	<0.001	4.9 [2.7, 8.8]	<0.001
Gender × Period				
Female × April 2021	1.3 [0.8, 2.2]	0.293	4.3 [1.6, 11.4]	0.003
Female × February 2021	1.1 [0.7, 1.8]	0.649	2.0 [0.8, 4.8]	0.120
Female × November 2020	0.9 [0.6, 1.5]	0.757	1.9 [0.8, 4.6]	0.131
Age group				
18–39/65+	7.1 [4.6, 11.1]	<0.001	4.0 [2.4, 6.6]	<0.001
40–64/65+	4.3 [2.8, 6.7]	<0.001	2.5 [1.5, 4.1]	<0.001
Age group × Gender				
18–39 × Female	0.4 [0.2, 0.7]	0.001	0.5 [0.2, 0.9]	0.018
40–64 × Female	0.4 [0.2, 0.7]	<0.001	0.8 [0.4, 1.4]	0.359
Education				
Up to junior high/postgraduate	2.7 [1.9, 3.9]	<0.001	4.3 [2.5, 7.4]	<0.001
Up to high school/postgraduate	2.1 [1.5, 3.0]	<0.001	2.6 [1.5, 4.3]	<0.001
University/postgraduate	1.8 [1.3, 2.5]	<0.001	1.6 [0.9, 2.7]	0.079
Child at home Yes/No	1.3 [1.1, 1.6]	0.015	0.9 [0.7, 1.3]	0.730

**^†^** RRR: relative risk ratio.

## Data Availability

Data are not available for sharing.

## Supplementary Materials

The following are available online at https://www.mdpi.com/article/10.3390/vaccines10030470/s1, Figure S1: Adjusted predictions of reporting safety concerns by gender and age group (among respondents unwilling to be vaccinated).

## References

[1] WHO Weekly Epidemiological Update on COVID-19—8 June 2021. https://www.who.int/publications/m/item/weekly-epidemiological-update-on-covid-19---8-june-2021.

[2] WHO (2020). Pandemic fatigue—reinvigorating the public to prevent COVID-19. Policy Framework for Supporting Pandemic Prevention and Management.

[3] World Health Organization Status of COVID-19 Vaccines within WHO EUL/PQ evaluation process. Guidance Document 02 March 2022. https://extranet.who.int/pqweb/sites/default/files/documents/Status_COVID_VAX_02March2022.pdf.

[4] Hodgson D., Flasche S., Jit M., Kucharski A.J., CMMID COVID-19 Working Group, Centre for Mathematical Modelling of Infectious Disease (CMMID) COVID-19 Working Group (2021). The potential for vaccination-induced herd immunity against the SARS-CoV-2 B.1.1.7 variant. Eurosurveillance.

[5] Bollyky T.J. (2021). U.S. COVID-19 Vaccination Challenges Go Beyond Supply. Ann. Intern. Med..

[6] MacDonald N.E. (2015). Vaccine hesitancy: Definition, scope and determinants. Vaccine.

[7] Hrynick T., Ripoll S., Schmidt-Sane M. (2020). Rapid Review: Vaccine Hesitancy and Building Confidence in COVID-19 Vaccination.

[8] Betsch C., Schmid P., Heinemeier D., Korn L., Holtmann C., Bohm R. (2018). Beyond confidence: Development of a measure assessing the 5C psychological antecedents of vaccination. PLoS ONE.

[9] Rosen B., Waitzberg R., Israeli A., Hartal M., Davidovitch N. (2021). Addressing vaccine hesitancy and access barriers to achieve persistent progress in Israel’s COVID-19 vaccination program. Isr. J. Health Policy Res..

[10] Fagerland M.W., Hosmer D.W., Bofin A.M. (2008). Multinomial goodness-of-fit tests for logistic regression models. Stat. Med..

[11] Lin C., Tu P., Beitsch L.M. (2020). Confidence and Receptivity for COVID-19 Vaccines: A Rapid Systematic Review. Vaccines.

[12] Edwards B., Biddle N., Gray M., Sollis K. (2021). COVID-19 vaccine hesitancy and resistance: Correlates in a nationally representative longitudinal survey of the Australian population. PLoS ONE.

[13] Paul E., Steptoe A., Fancourt D. (2021). Attitudes towards vaccines and intention to vaccinate against COVID-19: Implications for pub-lic health communications. Lancet Reg. Health Eur..

[14] Viswanath K., Bekalu M., Dhawan D., Pinnamaneni R., Lang J., McLoud R. (2021). Individual and social determinants of COVID-19 vac-cine uptake. BMC Public Health.

[15] Coe A.B., Elliott M.H., Gatewood S.B.S., Goode J.R., Moczygemba L.R. (2021). Perceptions and predictors of intention to receive the COVID-19 vaccine. Res. Soc. Adm. Pharm..

[16] Hammer C.C., Cristea V., Dub T., Sivela J. (2021). High but slightly declining COVID-19 vaccine acceptance and reasons for vaccine acceptance, Finland April to December 2020. Epidemiol. Infect..

[17] Lazarus J.V., Ratzan S.C., Palayew A., Gostin L.O., Larson H.J., Rabin K., Kimball S., El-Mohandes A. (2021). A global survey of potential acceptance of a COVID-19 vaccine. Nat. Med..

[18] (2021). Global Attitudes Towards a COVID-19 Vaccine. Imperial College London. https://www.imperial.ac.uk/media/imperial-college/institute-of-global-health-innovation/GlobalVaccineInsights.

[19] Kourlaba G., Kourkouni E., Maistreli S., Tsopela C.G., Molocha N.M., Triantafyllou C., Koniordou M., Kopsidas I., Chorianopoulou E., Maroudi-Manta S. (2021). Willingness of Greek general population to get a COVID-19 vaccine. Glob. Health Res. Policy.

[20] Maltezou H.C., Pavli A., Dedoukou X., Georgakopoulou T., Raftopoulos V., Drositis I., Bolikas E., Ledda C., Adamis G., Spyrou A. (2021). Determinants of intention to get vaccinated against COVID-19 among healthcare personnel in hospitals in Greece. Infect. Dis. Health.

[21] Papagiannis D., Rachiotis G., Malli F., Papathanasiou I.V., Kotsiou O., Fradelos E.C., Giannakopoulos K., Gourgoulianis K.I. (2021). Ac-ceptability of COVID-19 Vaccination among Greek Health Professionals. Vaccines.

[22] Sheeran P. (2002). Intention—Behavior relations: A conceptual and empirical review. Eur. Rev. Soc. Psychol..

[23] European Centre for Disease Prevention and Control (2021). Vaccine Tracker Stockholm: ECDC. https://vaccinetracker.ecdc.europa.eu/public/extensions/COVID-19/vaccine-tracker.html#uptake-tab.

[24] Reno C., Maietti E., Fantini M.P., Savoia E., Manzoli L., Montalti M., Gori D. (2021). Enhancing COVID-19 Vaccines Acceptance: Results from a Survey on Vaccine Hesitancy in Northern Italy. Vaccines.

[25] Wang K., Wong E.L.-Y., Ho K.-F., Cheung A.W.-L., Yau P.S.-Y., Dong D., Wong S.Y.-S., Yeoh E.-K. (2021). Change of Willingness to Accept COVID-19 Vaccine and Reasons of Vaccine Hesitancy of Working People at Different Waves of Local Epidemic in Hong Kong, China: Repeated Cross-Sectional Surveys. Vaccines.

[26] European Medicines Agency (2021). Astrazeneca’s COVID-19 Vaccine: Ema Finds Possible Link to Very Rare Cases of Unusual Blood Clots with Low Blood Plate-Lets. https://www.ema.europa.eu/en/news/astrazenecas-covid-19-vaccine-ema-finds-possible-link-very-rare-cases-unusual-blood-clots-low-blood.

[27] Bonioli M., McIsaac M., Xu L., Wuliji T., Diallo K., Campbell J. (2019). Gender Equity in the Health Workforce: Analysis of 104 Countries.

[28] Nguyen K.H., Srivastav A., Razzaghi H., Williams W., Lindley M.C., Jorgensen C., Abad A., Singleton J.A. (2021). COVID-19 Vaccination Intent, Perceptions, and Reasons for Not Vaccinating Among Groups Prioritized for Early Vaccination—United States, September and December 2020. MMWR Morb. Mortal. Wkly. Rep..

[29] Troiano G., Nardi A. (2021). Vaccine hesitancy in the era of COVID-19. Public Health.

[30] (2021). Education Is Now a Bigger Factor than Race in Desire for COVID-19 Vaccine. https://healthpolicy.usc.edu/evidence-base/education-is-now-a-bigger-factor-than-race-in-desire-for-covid-19-vaccine/.

[31] Baccolini V., Renzi E., Isonne C., Migliara G., Massimi A., De Vito C., Marzuillo C., Villari P. (2021). COVID-19 Vaccine Hesitancy among Italian University Students: A Cross-Sectional Survey during the First Months of the Vaccination Campaign. Vaccines.

