# Supplementary material for: Trends in COVID-19 Vaccination Intent, Determinants and Reasons for Vaccine Hesitancy: Results from Repeated Cross-Sectional Surveys in the Adult General Population of Greece during November 2020–June 2021
Source: Vaccines (Basel). 2022 Mar 18;10(3):470. doi: 10.3390/vaccines10030470 (PMC8950863; doi:10.3390/vaccines10030470)

Supplementary Figure S1. Adjusted predictions of reporting safety concerns by gender and age group (among respondents unwilling to get vaccinated)

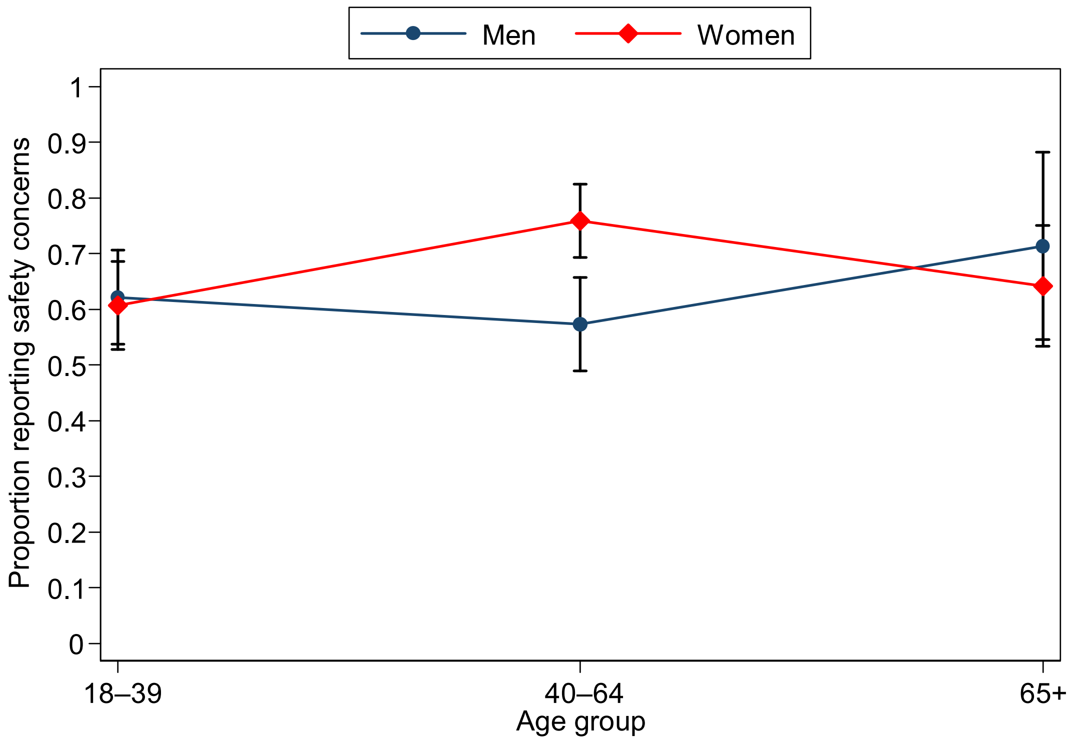

Supplement: Supplementary file 1 [file vaccines-10-00470-s001.zip › vaccines-1644226-supplementary.pdf]
